# Asymmetric Activation of ON and OFF Pathways in the Degenerated Retina

**DOI:** 10.1523/ENEURO.0110-24.2024

**Published:** 2024-05-14

**Authors:** Maya Carleton, Nicholas W. Oesch

**Affiliations:** ^1^Department of Psychology, University of California San Diego, La Jolla, California 92093; ^2^Department of Ophthalmology, University of California San Diego, La Jolla, California 92093; ^3^Neuroscience Graduate Program, University of California San Diego, La Jolla, California 92093

**Keywords:** circuit, electrical stimulation, retina, retinal degeneration, retinal prosthetics, synapse

## Abstract

Retinal prosthetics are one of the leading therapeutic strategies to restore lost vision in patients with retinitis pigmentosa and age-related macular degeneration. Much work has described patterns of spiking in retinal ganglion cells (RGCs) in response to electrical stimulation, but less work has examined the underlying retinal circuitry that is activated by electrical stimulation to drive these responses. Surprisingly, little is known about the role of inhibition in generating electrical responses or how inhibition might be altered during degeneration. Using whole-cell voltage–clamp recordings during subretinal electrical stimulation in the *rd10* and wild-type (*wt*) retina, we found electrically evoked synaptic inputs differed between ON and OFF RGC populations, with ON cells receiving mostly excitation and OFF cells receiving mostly inhibition and very little excitation. We found that the inhibition of OFF bipolar cells limits excitation in OFF RGCs, and a majority of both pre- and postsynaptic inhibition in the OFF pathway arises from glycinergic amacrine cells, and the stimulation of the ON pathway contributes to inhibitory inputs to the RGC. We also show that this presynaptic inhibition in the OFF pathway is greater in the *rd10* retina, compared with that in the *wt* retina.

## Significance Statement

Changes in circuit processing may have deleterious effects on vision restoration for patients with retinitis pigmentosa. Prior research has focused on feedforward excitatory drive and not the interactions between excitation and inhibition that comprise normal retinal function. This study demonstrates that retinal ganglion cells respond to electrical stimulation in three broad functional groups that correspond to their anatomical structure. We show that while both degenerated and *wt* retinas display the same three groups, the degenerated retina has an increased amount of OFF pathway presynaptic inhibition limiting their excitatory output to OFF ganglion cells.

## Introduction

Optogenetics and optoelectronic retinal prosthetics are both currently being pursued for vision restoration in patients with retinitis pigmentosa. Recently several new optogenetic therapies have entered clinical trials for retinitis pigmentosa (Drag et al., 2023); however, subretinal prosthetics are the only therapy in trials to restore lost vision in age-related macular degeneration (Pixium Vision, NCT03392324; Rubner et al., 2022), which is the most common form of retinal blindness (Bourne et al., 2020). This highlights the importance of continuing to pursue improvements in retinal prosthetics. Retinal prosthetics aim to recreate vision through implanted optoelectronic arrays to replace the function of photoreceptors and reactivate the remaining retinal circuitry with electrical stimulation. While implant technologies have been made available to patients, the quality of restored vision has been modest (Stingl et al., 2015, 2017; Palanker et al., 2020), and there are many questions remaining about the best strategies for vision restoration (Weiland et al., 2016; Damle et al., 2017).

The vertebrate retina is subdivided into two parallel pathways, the ON and OFF pathway (Schiller, 1992; Masland, 2001; Wässle, 2004). These pathways work together to encode increments and decrements of light, respectively. When light activates photoreceptors, ON bipolar cells (BCs) are depolarized, and OFF BCs are hyperpolarized. These parallel processing channels are maintained throughout the retina and lateral geniculate nucleus and into primary visual area V1. Subretinal prosthesis aim to restore purposeful vision, but a major concern surrounding retinal prosthetics is the ability to restore meaningful ON and OFF pathway responses. Past work has shown that ON and OFF retinal ganglion cells (RGCs) maintain their distinctive ON- and OFF-specific morphology following retinal degeneration (Marc et al., 2003; Margolis et al., 2008). Intriguingly, a few studies have shown that ON and OFF responses can be evoked during electrical stimulation in a degenerated retina (Sekhar et al., 2017; Ho et al., 2018, 2020). However, it remains unknown if these electrical ON and OFF responses originate in “true” ON and OFF RGCs or reflect anomalous signaling from artificial stimulation. Past work has shown that healthy ON and OFF pathways have stereotypical patterns of excitation and inhibition (Pang et al., 2003; Murphy and Rieke, 2006), which are maintained following degeneration in the rd1 mouse (Margolis et al., 2008); however, the circuit mechanisms that generate electrical ON and OFF responses and the role of inhibition remain completely unknown.

Inhibition arises from a diversity of amacrine cells to shape the spatial and temporal response properties of both RGCs and BCs. Inhibition can be further characterized as feedback (onto BCs), feedforward (onto ganglion cells), crossover (activity in one pathway suppresses elements of the other), or serial (onto other amacrine cells); for reviews, see Masland (2012) and Diamond (2017). It is plausible that prosthetic vision signals could activate any combination of inhibitory signaling. Ideally prosthetic vision restoration would activate inhibitory circuitry as in normal vision. However, electrical stimulation may directly and indiscriminately activate amacrine cells. Thus, to generate prosthetic visual signals with appropriate spatiotemporal characteristics, we must understand how it interacts with inhibitory signaling. Unfortunately, few studies have examined how inhibition in the degenerated retina is recruited in response to electrical stimulation (Margalit and Thoreson, 2006; Tsai et al., 2011; Walston et al., 2018).

To address these questions, we used whole-cell voltage–clamp techniques to measure electrically evoked excitatory and inhibitory synaptic inputs to RGCs in the *rd10* retina. We found three functional response patterns that corresponded to morphologically identified ON, OFF, and ON/OFF ganglion cells, with the prominent difference being in the excitation to inhibition ratio (E/I), where ON RGCs had an E/I ratio >1 and OFF cells had an E/I ratio <1. Interestingly, this is consistent with previous work for light-evoked inputs for a subset of RGCs (Pang et al., 2003; Murphy and Rieke, 2006) and with spontaneous inputs in rd1 mice (Margolis et al., 2008). Pharmacological dissection revealed that presynaptic feedback inhibition in the OFF pathway prevented OFF excitation due to a glycinergic amacrine cell and that direct inhibition in OFF RGCs originated in the OFF pathway.

## Materials and Methods

### Retinal explant and voltage-clamp recording

All experimental methods and animal care procedures were conducted in accordance with NIH guidelines and were approved by the University of California Institutional Animal Care and Use Committee. Thirty-four adult [age range, postnatal day (P)62–P296] *rd10* and eight C57BL mice of either sex were anesthetized with isoflurane, killed by cervical dislocation, and enucleated, and their retinas were dissected free and maintained in Ames’ medium oxygenated and equilibrated with 95% O_2_, 5% CO_2_. Retina pieces, ∼2 × 2 mm, were transferred to a custom recording chamber and placed over stimulating electrodes on the bottom of the custom recording chamber, ganglion cell side up. The chamber was placed under an upright microscope and perfused with Ames’ (Ames and Nesbett, 1981) media (United States Biological) at a rate of 4 ml/min at 35°C. RGCs were visualized and targeted using infrared (IR) differential interference contrast (DIC) video microscopy. Contact between the outer portion of the retina and the electrode array surface was also confirmed with microscopy. Stimulation was delivered on the nearest electrode to the target RGCs.

**Figure 1.**
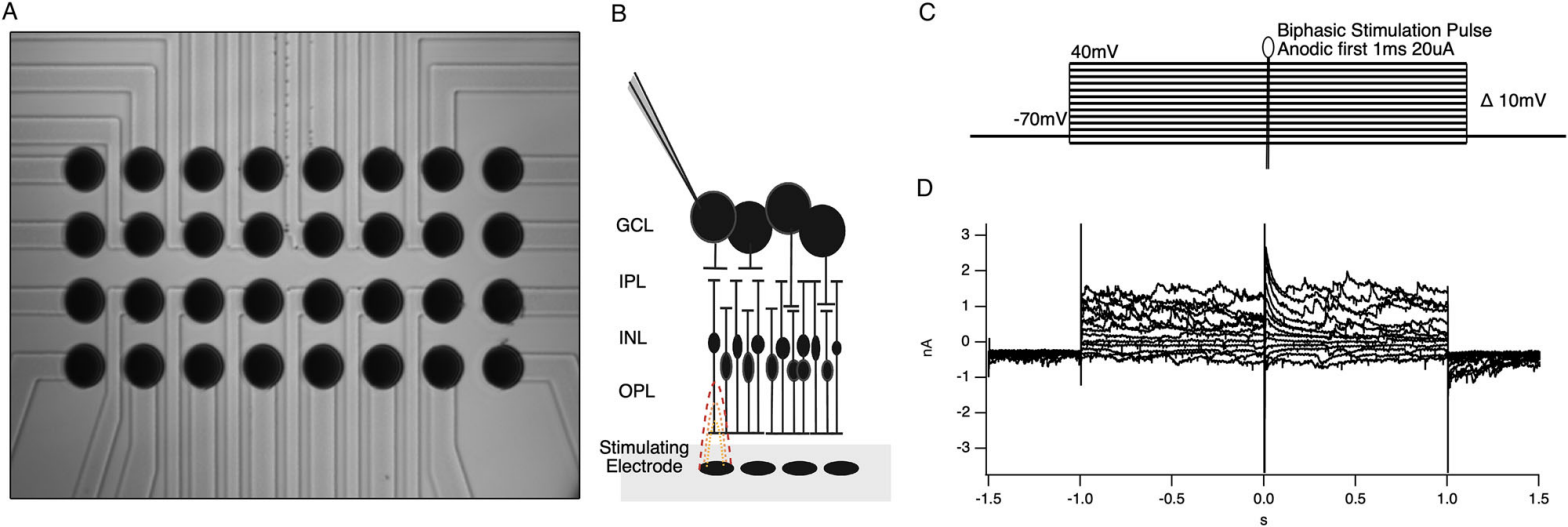
Experimental setup. ***A***, 4 × 8, 30 µm iridium oxide stimulating array. ***B***, Schematic of subretinal stimulation and current-clamp recording. ***C***, Voltage-step paradigm. Voltage from −80 to +40 mV. A biphasic, anodic-first, 1 ms, 20 µA stimulation pulse occurred at time 1.5 s, halfway through the voltage step. ***D***, Example currents recorded at each voltage step.

Sputtered iridium oxide film (SIROF) stimulating electrode arrays were fabricated as described previously on a borosilicate glass disk, which formed the bottom of the recording chamber (Damle et al., 2021). Briefly, the array consisted of a 4 × 8 grid of 30-µm-diameter SIROF electrodes spaced 50 µm apart from center to center. SIROF electrodes were formed by reactive DC sputtering to a thickness of 600 nm over indium tin oxide (ITO) traces. ITO traces terminated in gold contact pads at the edge of the disk for connection to a 32-channel RHS2000 stim and recording system (Intan Technologies). Charge-balanced, anodic–first, square, biphasic current pulses were generated on the RHS2000, triggered by our acquisition software, and delivered to an individual stimulating electrode nearest to the cell of interest. Arrays of 30-µm-diameter iridium oxide electrodes were used to stimulate the inner nuclear layer with 1 ms, 20 µA biphasic anodic-first pulses (Fig. 1*A*). Distances were measured using Scientifica LinLab2 and IR-DIC microscopy. The cell was positioned in the center of the field of view and marked as the coordinate zero point. The stage was then moved to the center of the stimulating electrode, and the *x* and *y* coordinates from LinLab2 were recorded.

Recording electrodes were pulled from borosilicate capillary glass to have a final resistance of 4–5 MΩ and filled with internal solution comprised of 90 mM CsCH3SO3, 20 mM TEA-Cl, 10 mM HEPES, 10 mM EGTA, 10 mM phosphocreatine disodium salt, 4 mM Mg-ATP, and 0.4 mM Na-GTP. Whole-cell voltage–clamp recordings were made from ganglion cells, and currents were recorded in voltage-clamp mode using a MultiClamp 700b (Molecular Devices) patch-clamp amplifier. Signals were filtered at 4 kHz (four-pole Bessel), digitized at 20 kHz with an ITC-18 (HEKA Elektronik) data acquisition board, and saved to a personal computer for off-line analysis using a custom acquisition software in Igor Pro 8 (WaveMetrics).

Pharmacological agents when used were added to the superfusate. Inhibition was blocked with a cocktail of 5 µM strychnine (Sigma-Aldrich), 5 µM SR-95531, and 50 mM TPMPA (Tocris Bioscience), to block glycine, GABA_A_, and GABA_C_ receptors, respectively. Gap junctions were blocked using 100 µM meclofenamic acid (MFA; Sigma-Aldrich), and rod BCs were hyperpolarized using 5 µM L-AP4 (Tocris Bioscience).

### Cell identification

**Figure 2.**
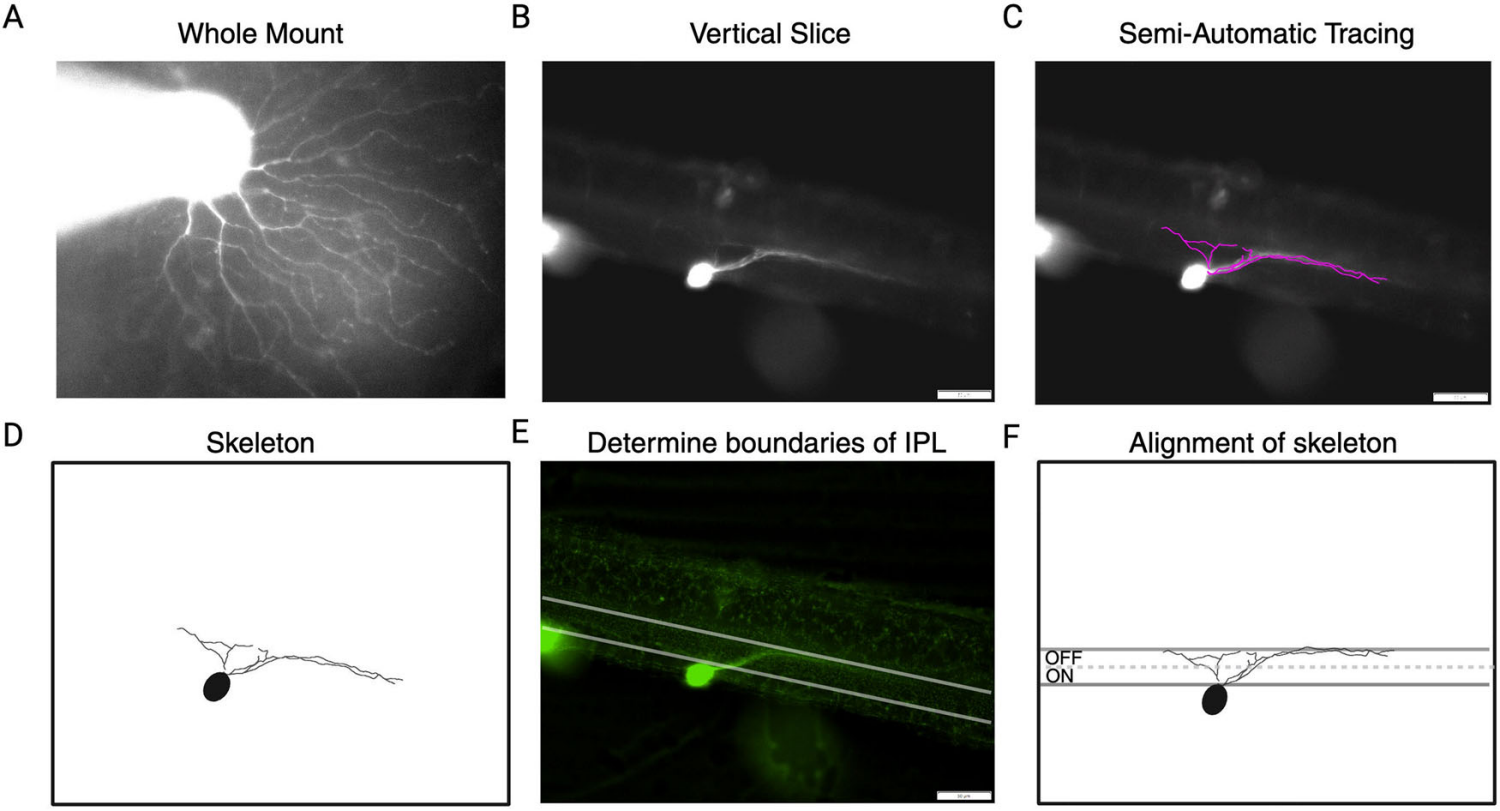
Analysis of cell morphology. ***A***, Image of example filled cell in whole mount. ***B***, The same retina vertically sliced at 200 µm to show dendritic stratification. ***C***, Semiautomatic tracing performed in NeuronJ. ***D***, Extracted skeleton from tracing. ***E***, Boundaries of IPL defined at the edges of the ganglion cell layer and inner nuclear layer. ***F***, The skeleton alighted to boundaries identified in (***E*)**.

RGCs were targeted for morphological analysis using an electrode containing internal solution with the addition of 2% Lucifer yellow (LY; Invitrogen). Cells were whole-cell voltage-clamped to record the functional response properties; during the recording, LY was diffused into the cell. The retina was then removed from Ames’ media and fixed in 4% paraformaldehyde in 0.1 M phosphate buffer (pH 7.4) for 45 min. Retinas were then washed in 0.1 M phosphate buffer for 25 min before being placed in 3% agarose (Sigma-Aldrich). Agarose blocks containing the retina were oriented vertically on a PELCO easiSlicer (Ted Pella) and sliced at 200 µm width. The sliced retina was plated and imaged on an epifluorescence microscope (Olympus, BX43). The inner and outer boundaries of the inner plexiform layer (IPL) were determined by the ganglion cell layer and the inner nuclear layer (Fig. 2). Cells were classified as ON, OFF, or ON/OFF based on dendrite depth within the IPL. The top boundary was denoted as 0% depth, and the bottom boundary was 100% depth; based on prior methods from Margolis and Detwiler (2007), we used 50% as the ON/OFF subliminal boundary. Dendritic arbors were traced using NeuronJ (ImageJ, National Institutes of Health).

### Analysis

Data processing and statistical analyses were performed in Igor Pro 8. Conductance analysis was performed in a similar method to previous studies (Borg-Graham, 2001; Oesch and Taylor, 2010). Stimulus-evoked responses were recorded at 13 holding potentials (from −80 to +40 in 10 mV steps, Fig. 1*B*,*C*). I–V relationships were measured at 5 ms intervals for the duration of the voltage step, relative to the baseline in the preceding 500 ms. Voltage series were repeated two times and averaged. Synaptic currents were assumed to arise from a sum of linear excitatory and inhibitory synaptic inputs. Excitation is mediated by nonselective cation channels with a reversal potential of *V_e_* =  0 mV, while inhibition is mediated by chloride channels with a reversal potential of *V_i_* = −52 mV. Each I–V was fit with a line between −80 and −40 mV where the I–V relation was most linear. The slope and intercept were determined from the fit, thus producing a discrete measurement of conductance at every point.

## Results

**Figure 3.**
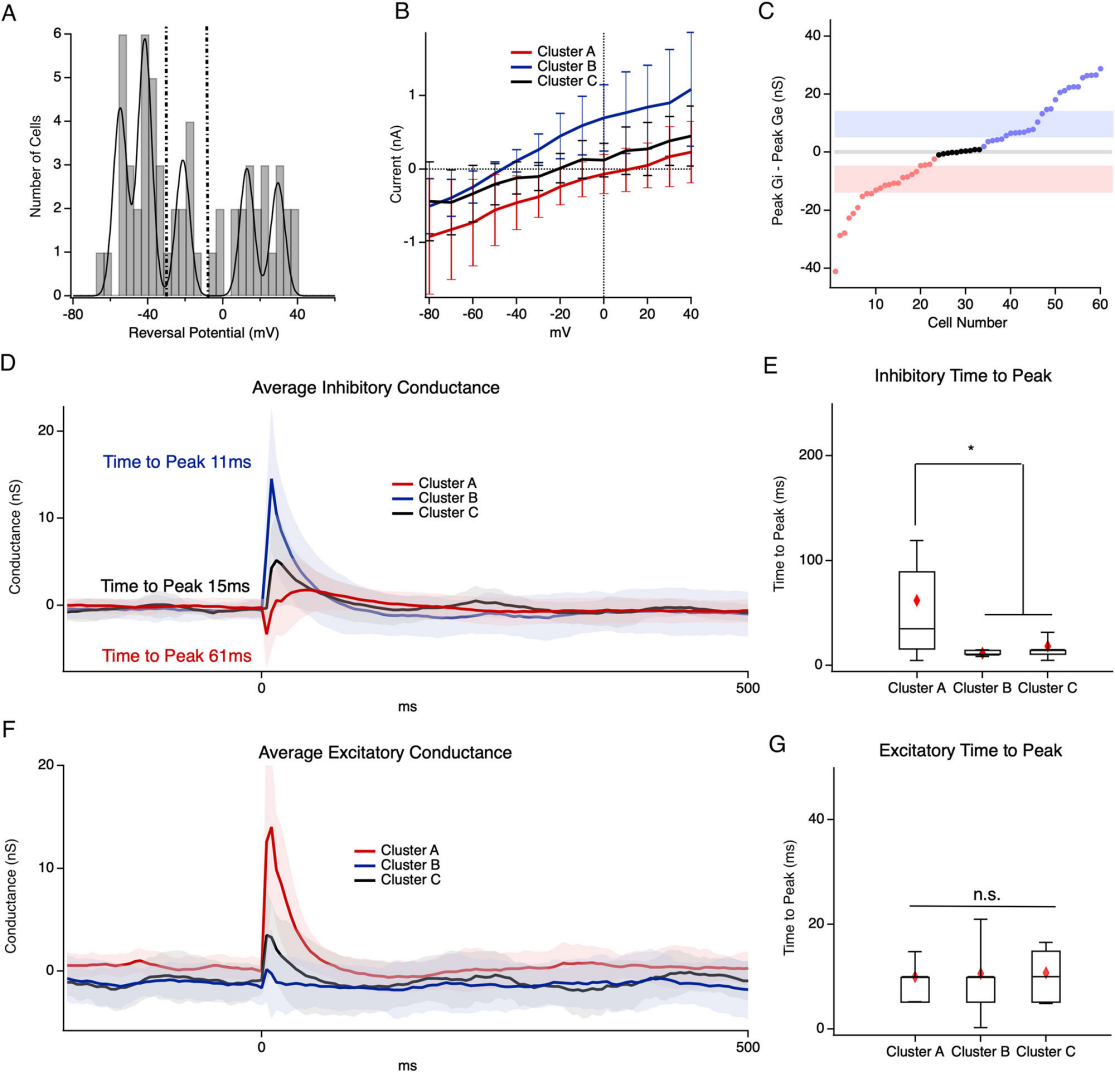
Functional grouping for 60 cells. ***A***, Cells were divided into three groups based on the histogram of their reversal potential; dotted lines represent boundaries for individual groups. ***B***, Average I–V curves for each cluster. Error bars represent 1 SD. Cluster A (red line, *n* = 23), Cluster B (blue line, *n* = 27), and Cluster C (black line, *n* = 10). ***C***, Peak Gi–peak Ge for cells from ***B***; shading represents 1 SD for each group. ***D***, Average inhibitory conductance traces for each cluster. Shading represents SEM. ***E*,** Box plot for excitatory conductance time to peak values for Cluster A, Cluster B, and Cluster C. ***F***, Average excitatory conductance traces for each cluster. ***G***, Same as ***E*** but for inhibitory conductance. Error bars represent 1 SD; red diamond is the mean; * indicates significance *p* < 0.05.

Inhibition performs many functions in the retina to process signals, such as center-surround organization (Cook and McReynolds, 1998; Taylor, 1999; Flores-Herr et al., 2001), gain modulation (Sagdullaev et al., 2006; Oesch and Diamond, 2019; Nagy et al., 2021), decorrelation (Franke et al., 2017), and mediating information transfer between ON to OFF pathways (Molnar et al., 2009; for review, see Diamond and Lukasiewicz, 2012). The role of inhibition in response to electrical stimulation or how inhibition might function in the *rd10* retina is not yet clear (Cameron et al., 2013; Walston et al., 2018). To examine excitatory and inhibitory network responses to electrical stimulation in the *rd10* retina, we used whole-cell voltage–clamp techniques to measure stimulation-evoked synaptic conductance. We recorded electrical stimulation-evoked currents from 60 cells over a range of holding potentials between −80 and +40 mV. We found that reversal potentials for stimulation-evoked currents were not normally distributed (Shapiro–Wilk test; *p* = 0.0006) and had three distinct modes (Fig. 3*A*). One mode was dominated by excitatory conductance, one dominated by inhibitory conductance, and one weaker intermediate mode with an equal mix of both excitation and inhibition. We divided the distribution into three groups centered around these modes by setting boundaries at the minima between the peaks of the multipeak Gaussian fit. Points where the fit decreased to zero were considered boundaries. Group 1 ranged from +40 to −10 mV (*n* = 23), Group 2 ranged from −80 to −35 mV (*n* = 27), and Group 3 ranged from −35 to −10 mV (*n* = 10). Plotting the average I–V and SEM for each mode showed distinct and nonoverlapping I–V relations for each group, which had roughly similar total conductance despite distinct reversal potentials (Fig. 3*B*). When we compared the excitatory and inhibitory conductance for the three groups identified by the distribution of I–Vs, we found that Group 1 excitation was significantly larger than inhibition and Group 2 cells had inhibition that was significantly larger than excitation (*p* = 5.9 × 10^−7^, *t* test; *p* = 3.8 × 10^−9^, *t* test). When we plot the difference between peak inhibitory and excitatory conductance, we find the SD of each group does not overlap with the neighboring groups (Fig. 3*C*).

Given the large sample of RGCs within each group, it is likely that each group is itself comprised of a heterogeneous population of RGC subtypes, but we reasoned that these broad E/I groups may share some properties within groups. Cells in Groups 2 and 3 displayed fast inhibition with a similar time to peak (*p* = 0.82, Tukey’s post hoc test) and little variation in the population of waveforms. In contrast, Group 1 cells displayed a significantly slower time to peak for inhibitory conductance and significantly more variability in responses, possibly indicating a diversity of network-mediated inhibitory effects contributing to Group 1 inhibition (*p* = 2.05 × 10^−5^, ANOVA; *p* < 0.05, Bartlett’s variance test; Fig. 3*D,E*). Although Group 2 cells had very little excitatory conductance, the kinetics of excitation were similar for all groups (Fig. 3*F*,*G*).

### Morphological and functional classification

**Figure 4.**
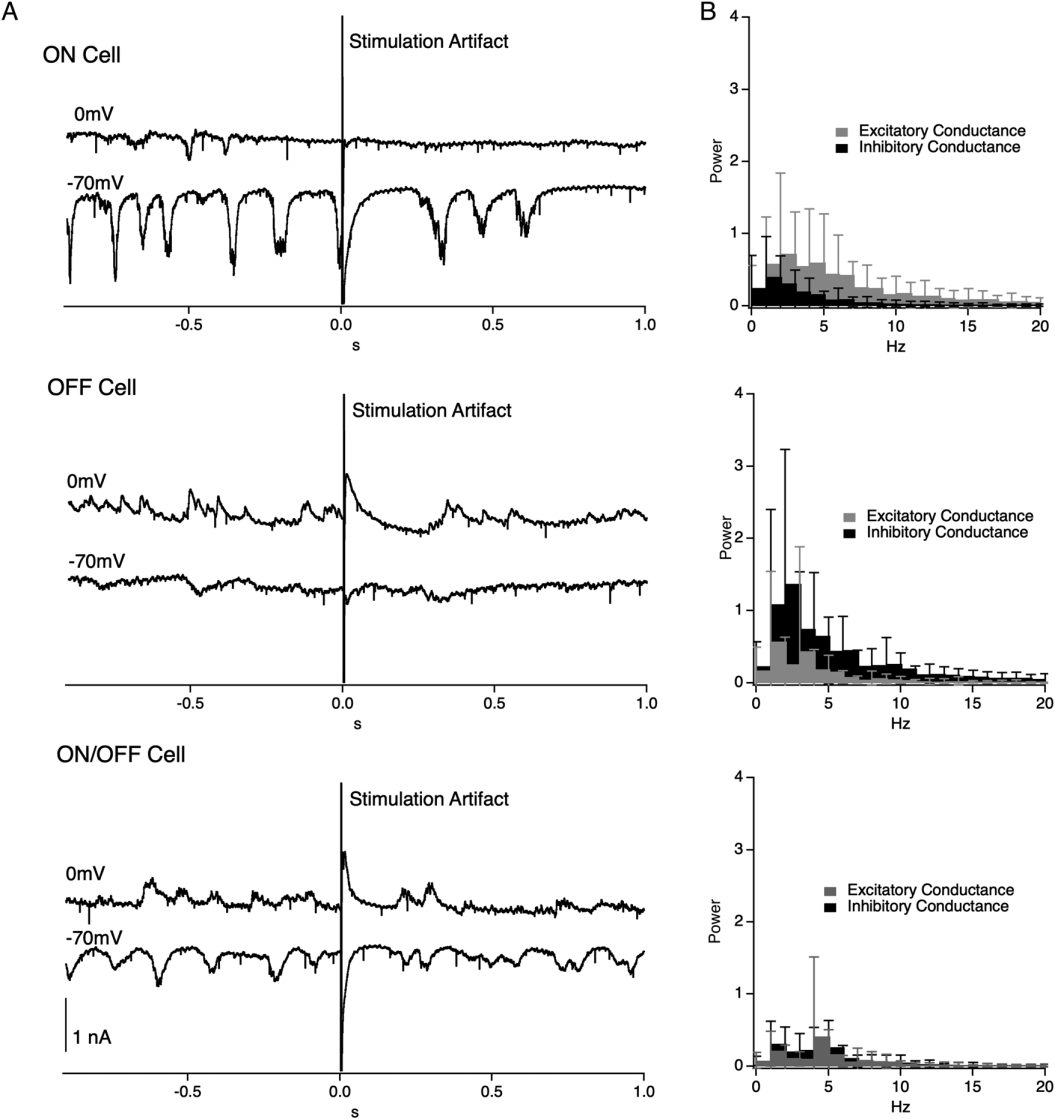
Oscillatory currents and conductance. ***A***, Example current traces for a putative ON (top), OFF (middle), and ON/OFF (bottom) cell at holding potentials of 0 mV and −70 mV. ***B***, Average excitatory and inhibitory conductance PSD for all ON (top) and OFF (bottom) cells. Gray represents excitatory conductance, and solid black represents inhibitory conductance; error bars are 1 SD.

Previous work suggested that similar patterns of light-evoked E/I balance correspond to ON and OFF functional groups for certain subtypes of RGCs (Pang et al., 2003; Murphy and Rieke, 2006; Roska et al., 2006; Margolis and Detwiler, 2007), and we hypothesized that electrically evoked E/I Groups 1, 2, and 3 may correspond to ON, OFF, and ON/OFF RGCs, respectively. Margolis et al. (2008) found a similar pattern of E/I balance in the spontaneous synaptic inputs between morphologically identified ON and OFF cells in the rd1 mouse; however, the E/I ratio of stimulation-evoked synaptic inputs was not measured for light or electrical responses. Whether these patterns of stimulation-evoked excitation and inhibition hold true following degeneration has not yet been shown. We examined spontaneous excitatory and inhibitory inputs to RGCs in our groups to see if stimulation-evoked E/I groups were consistent with previous findings for spontaneous activity. We found an ∼10-fold difference in the power of inhibitory conductance between Group 1 and Group 2 RGCs in the frequency range of 2–12 hz (Fig. 4*B*); consistent with stimulation-evoked E/I, groups corresponded to ON and OFF RGCs. This indicates that the E/I balance of electrically evoked synaptic inputs in Group 1 and 2 RGCs is consistent with previous findings of a unique E/I balance for degeneration-induced spontaneous synaptic inputs in ON and OFF RGCs, respectively.

**Figure 5.**
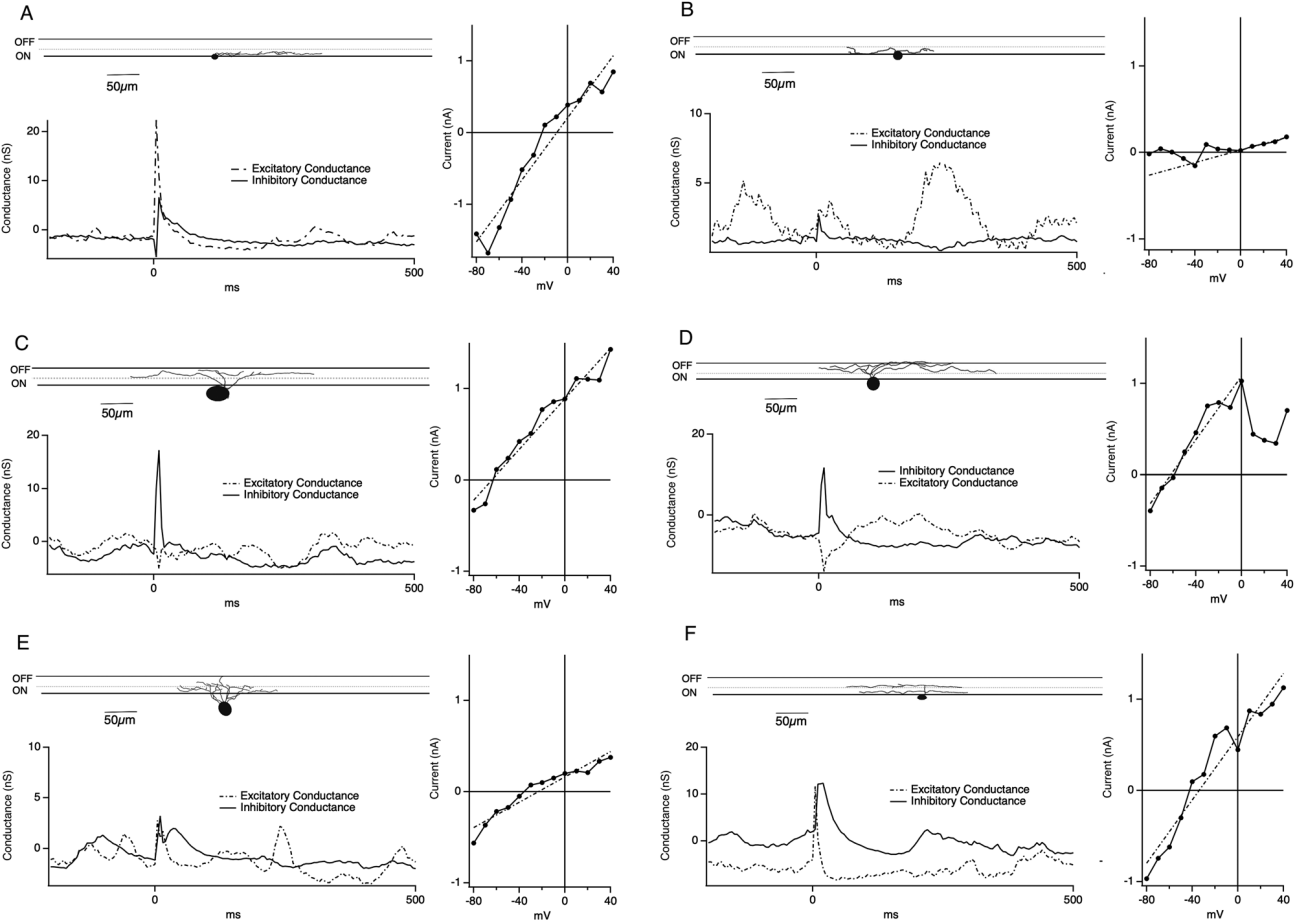
Morphological analysis of recorded RGCs. RGCs were filled with LY during the recording, fixed and sliced vertically to image dendritic termination in the IPL. ***A***, Top: RGC dendrites terminating in the ON layer of the IPL. Left: example excitatory and inhibitory conductance trace from the filled cell. Right: I–V curve from the filled cell. ***B***, Same as ***A*** for a different filled RGC with dendrites terminating in the ON layer. ***C***, Top: RGC dendrites terminating in the OFF layer of the IPL. Left: example excitatory and inhibitory conductance trace from the filled cell. Right: I–V curve from the filled cell. ***D***, Same as ***C*** for a different filled RGC with dendrites terminating in the OFF layer. ***E***, Top: RGC dendrites terminating in the ON and OFF layer of the IPL. Left: example excitatory and inhibitory conductance trace from the filled cell. Right: I–V curve from the filled cell. ***F***, Same as ***E*** for a different filled RGC with dendrites terminating in the ON and OFF layer. In all panels, black traces represent inhibitory conductance, and gray dotted traces represent excitatory conductance.

To determine if there was a correlation between physiological groups and ON and OFF morphological structure, we filled cells with LY and recovered RGC morphology postrecording in a subset of recordings (*n* = 12, at least three from each group, Fig. 5). For all filled cells, the recovered cell morphology matched the predicted functional group: Cluster A corresponded to ON cells, Cluster B corresponded to OFF cells, and Cluster C corresponded to ON/OFF cells. Thus, both morphological and functional analyses of clusters indicate that the three electrical stimulation-evoked response patterns we observed are representative of the three main classes of RGCs, ON, OFF, and ON/OFF.

Our results demonstrate three separable groups based on E/I with ON/OFF RGCs occupying an intermediate E/I group. Interestingly, although past work only focused on a few subtypes of RGCs (Pang et al., 2003; Murphy and Rieke, 2006), our large sample of RGC recordings indicates that this stereotypical E/I grouping may apply to many subtypes of RGCs (Roska et al., 2006), although we did not confirm the individual subtype identity.

### Mechanisms underlying E/I ON and OFF pathway differences

Given that subretinal electrical stimulation is expected to activate ON and OFF BCs to the same extent (Farzad et al., 2023; but see Twyford et al., 2014), we were surprised to find that OFF RGCs received little excitatory input. There are three primary possibilities to account for this: (1) stimulation-evoked inhibition suppresses OFF BCs, (2) OFF BCs may be hyperpolarized during degeneration and are unable to reach threshold, or (3) OFF BCs are not excited by electrical stimulation.

**Figure 6.**
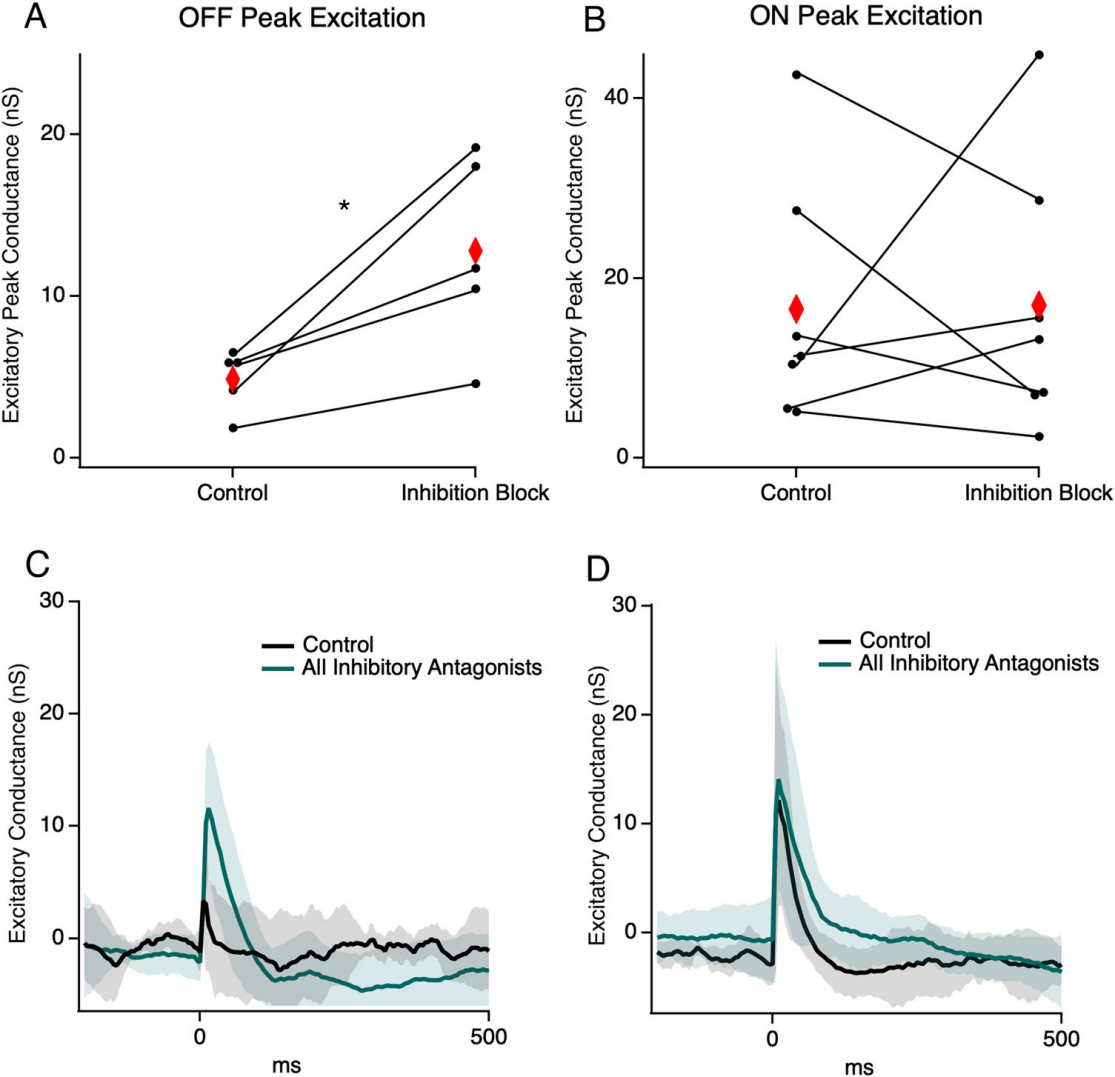
Change in excitatory conductance with application of inhibitory antagonists SR-95531, TPMPA, and strychnine. ***A***, A pairwise plot of peak excitatory conductance between the control and inhibition block groups in OFF cells (*n* = 5). Red diamonds indicate the mean. ***B***, Same as ***A*** but for ON cells (*n* = 7). ***C***, ***D***, Average excitatory conductance trace for cells from the panels above; shading represents 1 SD. * indicates significance *p* < 0.05.

Presynaptic inhibition of BC terminals is a well-described inhibitory motif in the retina. We therefore hypothesized that stimulation may activate some amacrine cells, either directly or indirectly, to limit the OFF BC output during stimulation. To test this possibility, we recorded from RGCs during the GABA_A_, GABA_C_, and glycine receptor block with SR-95531, TPMPA, and strychnine, respectively. Consistent with inhibitory suppression of the OFF BC output, OFF RGC excitatory conductance was significantly increased in the presence of inhibitory antagonists from 1.28 ± 5.96 to 4.88 ± 1.88 nS (*p* = 0.02; paired *t* test; *n* = 5; Fig. 6*A*,*C*). For ON RGCs, the average excitatory conductance did not change, although individual cell responses were quite heterogeneous with some showing an increase and some showing a decrease in excitation (Fig. 6*B*,*D*). This suggests that ON BCs may receive more diverse inhibitory signaling than OFF BCs, where some are suppressed by presynaptic inhibition and in others inhibition may function to increase gain (Sagdullaev et al., 2006; Eggers et al., 2007, p. 200; Oesch and Diamond, 2019; Nagy et al., 2021). In contrast, OFF cells showed a consistent increase in excitatory conductance under inhibitory blockade, indicating that presynaptic inhibition plays a uniform role in gating the excitatory output of OFF BCs to OFF RGCs, at least during electrical stimulation.

**Figure 7.**
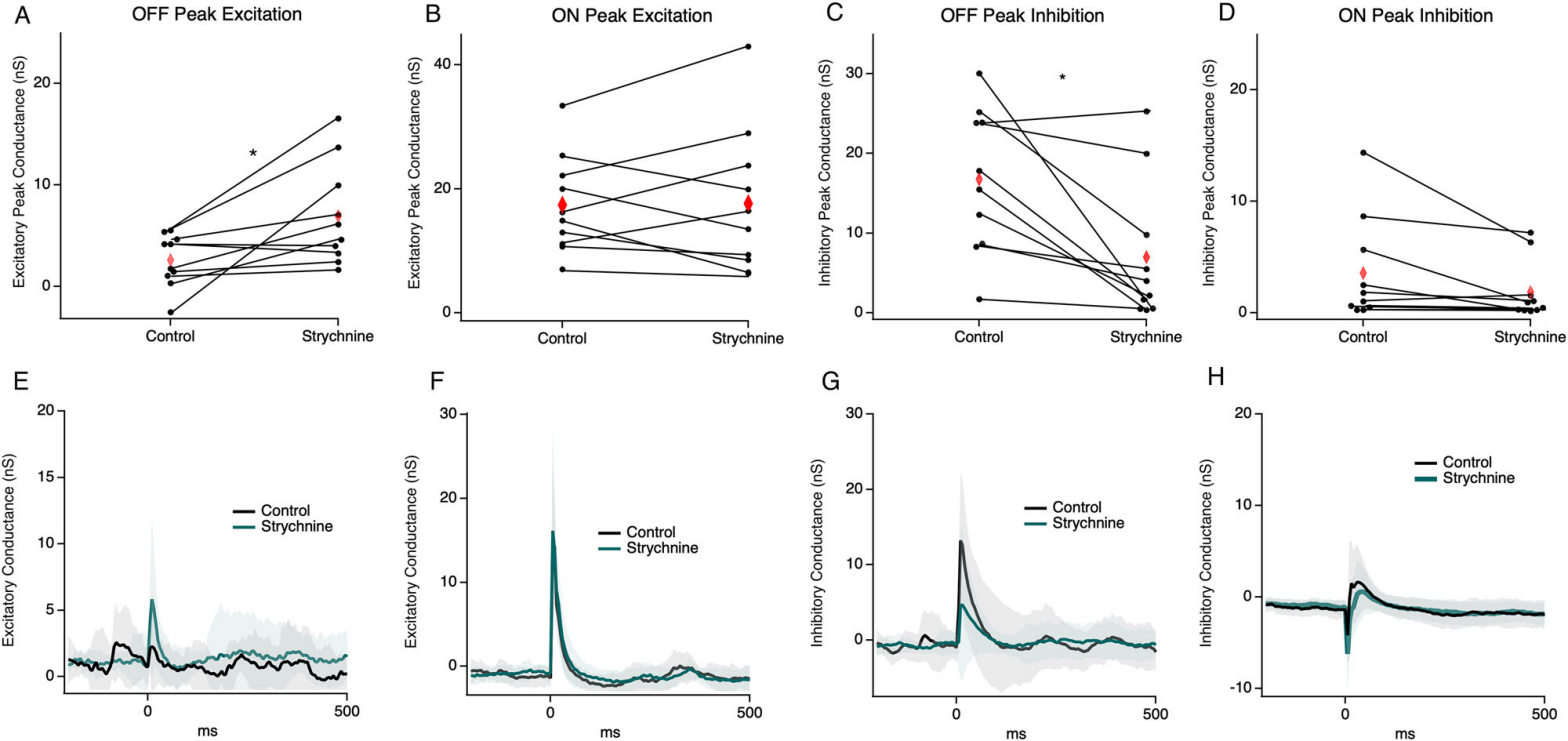
Change in conductance in the glycine block. ***A***, A pairwise plot of peak excitatory conductance between control and strychnine in OFF cells (*n* = 10). Red diamonds indicate the mean. ***B***, Same as ***A*** but for ON cells (*n* = 10). ***C***, A pairwise plot of peak inhibitory conductance between the control and strychnine in OFF cells (*n* = 10). ***D***, Same as ***C*** but for ON cells (*n* = 10). ***E–H***, Average conductance traces for cells from the subsequent panels above; shading represents 1 SD. * indicates significance *p* < 0.05.

Given that the glycinergic AII amacrine cell (AII) is well known to inhibit the OFF bipolar output during ON pathway excitation, as in the primary night vision pathway (Murphy and Rieke, 2008; Münch et al., 2009; Tian et al., 2010), we hypothesized that AIIs mediate OFF BC inhibition during stimulation. To examine this, we applied the glycine receptor antagonist strychnine. We found that on average OFF cell excitatory conductance increased, although to a smaller degree than for the complete inhibition block, from 2.61 ± 2.6 to 6.93 ± 4.98 nS (Fig. 7*A*,*E*; *p* = 0.018; paired *t* test; *n* = 10). Most OFF RGCs showed this effect (8 out of 10), indicating that most, but not all, presynaptic gating of OFF excitation is mediated by glycinergic amacrine cells. ON cells displayed a heterogeneous mix of responses during the glycine block but on average had the same amount of peak excitatory conductance, similar to results for the complete inhibition block (Fig. 6*B*,*F*).

Many OFF RGCs receive direct inhibitory inputs from AII amacrine cells (Manookin et al., 2008; Murphy and Rieke, 2008; Münch et al., 2009; van Wyk et al., 2009; Anderson et al., 2011) or other glycinergic amacrine cells (Buldyrev et al., 2012). For most OFF RGCs (6 out of 10), direct inhibitory conductance also decreased in strychnine compared with that in the control (Fig. 7*C*,*G*; 16.7 ± 9.03 to 6.98 ± 8.82 nS; *p* = 0.009; paired *t* test; *n* = 10). These six RGCs were also among the cells that showed glycine-mediated gating of excitation, leaving two cells which showed glycine-mediated presynaptic modulation of excitation, but no direct glycinergic inputs. This indicates that most OFF RGCs that receive direct glycinergic input also receive inputs from OFF BC subtypes that are modulated by glycinergic inputs. For ON RGCs, inhibition was not significantly changed (*p* = 0.33, *t* test) in the glycine receptor blockade with no distinct pattern between the direction of excitation and inhibition between the control and strychnine (Fig. 6*D*,*H*; 3.56 ± 9.03 nS to 1.85 ± 2.63 nC).

### The ON pathway shapes OFF cell inhibition

**Figure 8.**
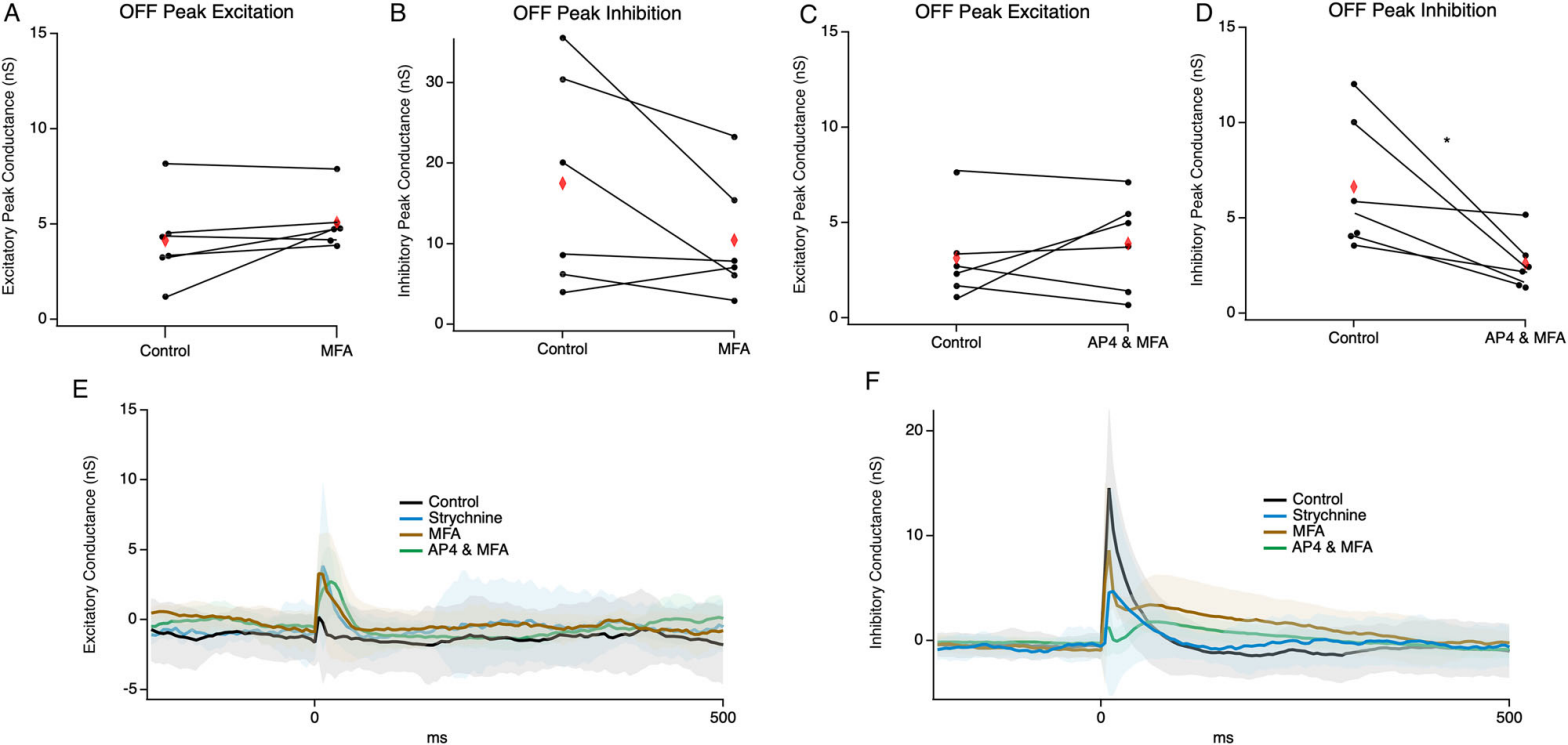
Contributions of the ON pathway to OFF cell inhibition and excitation. ***A***, A pairwise plot of peak excitatory conductance between control and MFA groups in OFF cells (*n* = 6). Red diamonds indicate the mean. ***B***, Same as in ***A*** but for inhibitory conductance. ***C***, A pairwise plot of peak excitatory conductance between control and MFA and AP4 groups in OFF cells (*n* = 6). ***D***, Same as in ***C*** but for inhibitory conductance. ***E***, Average excitatory conductance trace for cells from each group; shading represents 1 SD. ***F***, Same as ***E*** but average inhibitory conductance. * indicates significance *p* < 0.05.

Our results show that glycinergic inhibition mediates both direct inhibitory inputs to a majority of OFF RGCs and gates excitatory inputs to the same subset of OFF RGCs. Although a variety of glycinergic amacrine cell subtypes could be involved in both pre- and postsynaptic inhibition (Buldyrev et al., 2012), the AII amacrine cell can potentially mediate both effects and has been heavily implicated in both functions (Manookin et al., 2008; Münch et al., 2009; van Wyk et al., 2009; Buldyrev and Taylor, 2013; McLaughlin et al., 2021); for review, see Demb and Singer (2012). Considering this, we hypothesized that crossover signals from the ON pathway may be mediating inhibitory signaling to both OFF BC terminals and OFF RGCs. To examine the contributions of the ON cone BCs and rod bipolar-mediated inputs that would drive AII-mediated crossover inhibition, we first applied the gap junction blocker MFA, which should block signals that arise in the ON cone BCs. OFF RGC excitatory inputs remained unchanged in the MFA block (Fig. 8*A*,*E*). However, during the gap junction block, inhibitory inputs to OFF RGCs were decreased but were not significantly different from the control (Fig. 8*B*,*F*, paired *t* test, *p* = 0.1). Together this indicates that activation of ON cone BC inputs alone does not significantly contribute to presynaptic inhibition of OFF BCs or direct inhibition of OFF RGCs. Although MFA is often used to dissect this circuit (Liang and Freed, 2010), it is important to note that there are numerous gap junctions throughout the retina and the effect of MFA be mediated by effects at other gap junctions or even off-target effects at other receptor systems (Lee and Wang, 1999; Peretz et al., 2005).

Stimulation of rod bipolar cells (RBCs) could also be responsible for driving AII activity. Since we cannot block the RBC-mediated glutamatergic drive to AIIs without blocking excitatory inputs to OFF RGCs, we reasoned that we might reduce their contribution by hyperpolarizing RBCs with the mGluR6 agonist AP4 (Slaughter and Miller, 1983) in addition to MFA. It is important to note that this may not completely eliminate the RBC to AII input, because electrical stimulation may still be sufficient to evoke RBC release in the presence of AP4.

Despite this caveat, we observed a 50% reduction in inhibitory inputs to OFF RGCs when blocking both gap junctions and attenuating RBC input. This indicates that the RBC to the AII pathway does contribute to direct inhibition of OFF RGCs (*p* = 0.03, paired *t* test, Fig. 8*D*), thus implicating the AII. We did not, however, observe a significant increase in excitatory conductance in OFF RGCs (Fig. 8*A*,*C*). These results indicate that separate populations of glycinergic amacrine cells may contribute to pre- and postsynaptic OFF RGC inhibition and that postsynaptic inhibition involves a crossover inhibitory pathway. Alternatively, it is possible that one cell such as the AIIs mediates both types of inhibition, but its output synapses must have different input–output functions for bipolar and ganglion cell synapses, which differentially process information routed to these two targets. While our data supports the AII as contributing to crossover signals, we cannot rule out other amacrine cells or disinhibition of amacrine cells also playing a role in this phenomenon.

### Remnant cone effect on E/I balance

**Figure 9.**
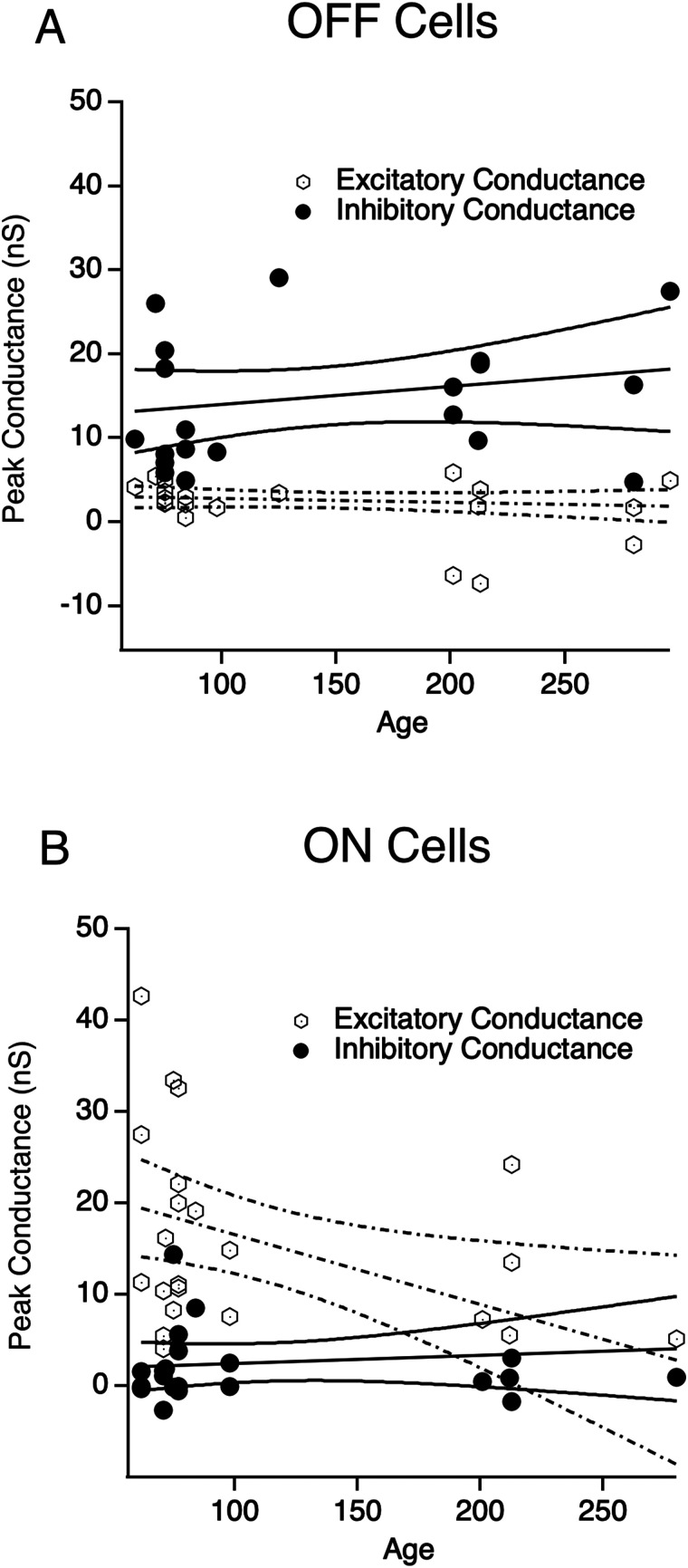
Peak excitatory and inhibitory conductance as a function of animal age. ***A***, OFF cell excitatory (open circle) and inhibitory (closed circle) peak conductance plotted against age**.** Linear regression plots for each. ***B***, Same as ***A*** for ON cells.

We have interpreted these findings as resulting from direct stimulation of bipolar and/or amacrine cells; however, recent work has demonstrated that cone inner segments remain intact and light responsive in the *rd10* mouse for a significant amount of time (around p67) after outer segment degeneration (Ellis et al., 2023). Given that electrical cone stimulation would increase glutamate release, increasing OFF pathway activity and decreasing ON activity, it is not obvious how cone activation could explain our results. Regardless, it may be possible that some residual cone inner segments remain and contribute to abnormal signaling in the ON or OFF pathways. Since cones eventually fully degenerate, the influence of the putative residual cone-mediated responses should decrease with age if they contribute. We plotted peak excitatory and inhibitory conductance against age for ON and OFF RGCs. For OFF RGCs, we found that age had no effect on peak excitatory or inhibitory conductance (Fig. 9*A*). In ON RGCs, there was a significant decrease in the excitatory conductance (Fig. 9*B*, Pearson's test, *R* = −0.475). This could indicate that residual cone inner segments provide some contribution to ON excitation; alternatively, this could be due to other disease-mediated changes in ON pathway synaptic transmission (Puthussery and Taylor, 2010). Given that Ellis et al. showed that residual cones should be almost completely gone by our youngest ages, we favor the latter hypothesis, although we cannot distinguish between these two possibilities here.

### E/I groupings are present in both the *rd10* and *wt* retina

**Figure 10.**
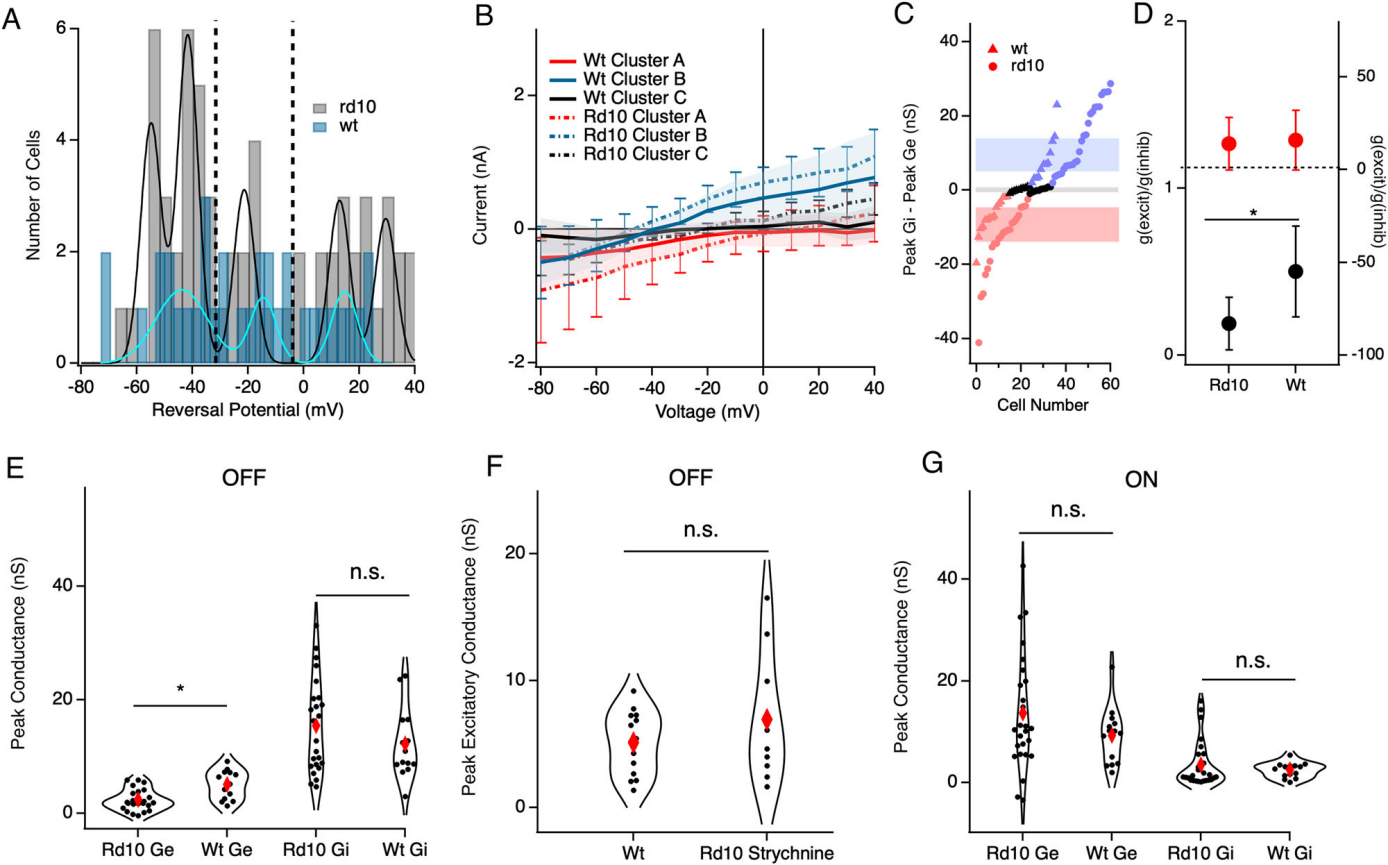
Functional grouping for *rd10* versus *wt* ON and OFF cells. ***A***, Cells clustered into three groups based on the histogram of their reversal potential; dotted lines represent cutoff points for individual groups. ***B***, Average I–V curves for each cluster. Shading represents 1 SD. Cluster A (red line, *n* = 15), Cluster B (blue line, *n* = 12), and Cluster C (black line, *n* = 10); dotted lines represent the average I–Vs for *rd10* groups in Figure 2. ***C***, Peak Gi–peak Ge for cells from ***B***; triangles indicate *wt*, and dots indicate *rd10* cells; shading represents 1 SD for each group. ***D***, E/I ratios for OFF cells (black) and ON cells (red); dotted line shows 1 on the right axis. ***E***, Violin plots showing the peak excitatory and inhibitory conductance in OFF cells of the *wt* and *rd10* retina. ***F***, A violin plot comparison of *wt* Ge versus *rd10* Ge in strychnine application. ***G***, Same as ***D*** but for ON cells. Red diamond is the mean; * indicates significance *p* < 0.05.

Past work has demonstrated E/I ratios in the wild-type (*wt*) retina consistent with the E/I balance we observed in the *rd10* retina, but only in a few identified subtypes. It may be possible that the correlation between E/I ratios extends broadly across multiple subtypes in the *wt* retina, as our data for *rd10* suggest, or it may be that the general relationship between E/I ratio across ON and OFF RGC class is unique to retinal degeneration. To address this question, we measured E/I balance for electrical stimulation in the *wt* retina as we did for *rd10* RGCs. We found *wt* cells had a distribution of reversal potentials similar to those observed in *rd10* corresponding to three distinct groups (Fig. 10*A–C*). These groups once again showed distinct I–V relations, with reversal potentials consistent with *rd10* for Groups 1 and 3; however, *wt* Group 2 cells had a more positive reversal potential than *rd10* Group 2 cells (Fig. 10*B*). Consistent with this observation, when we compared the E/I ratio between *rd10* and *wt* OFF cells, there was a significant increase in the E/I ratio from *rd10* to *wt* (Fig. 10*D*; *p* = 0.0009; *t* test). ON cells showed no difference in E/I ratio between *rd10* and *wt* (Fig. 10*D*).

To examine the source of this difference in reversal potential, we compared the magnitude of excitatory and inhibitory conductance between *rd10* and *wt* OFF RGCs. We found that excitatory conductance was significantly greater (*p* = 0.001, *t* test) in *wt* OFF RGCs compared with that in *rd10* OFF RGCs, but there was no significant difference in peak inhibitory conductance (Fig. 10*E*, *p* = 0.18, *t* test). Therefore, greater excitation, not reduced inhibition, drives the more positive reversal potential and E/I ratio closer to 1 for OFF RGCs in *wt* compared with those in *rd10*. Given that we observed significant presynaptic inhibition of excitatory OFF inputs to RGCs in *rd10* retina, we reasoned that this difference may be due to increased presynaptic inhibition in *rd10* OFF circuit. To test this hypothesis, we compared the magnitude of excitatory conductance in *wt* OFF RGCs to the magnitude of OFF RGC conductance when we blocked glycine, to relieve presynaptic glycinergic inhibition to the OFF BCs. When glycinergic inhibition was blocked, the difference between *wt* and *rd10* OFF RGCs was eliminated (Fig. 10*F*, *p* = 0.3, *t* test). For ONs, we found no significant differences between ON excitatory and inhibitory conductance (Fig. 10*G*, *p* = 0.9, *t* test) showing that the excitation–inhibition balance of the ON pathway is not disrupted in degeneration. Taken together, this indicates abnormal inhibitory signaling in the *rd10* OFF pathway, specifically for a subset of circuits which provide presynaptic inhibition, but not direct inhibition to OFF RGCs.

## Discussion

Here we provide evidence that electrical stimulation differentially modulates responses in the ON and OFF pathways. Similar to light stimulation, electrically evoked inputs for ON cells have an E/I ratio >1 with excitation dominating, while OFF cells had an E/I ratio <1 weighted toward inhibition. For ON cells, the time course of inhibitory conductance was variable suggesting multiple diverse sources of inhibitory inputs, while OFF cells had fast inhibitory conductance with little variance in kinetics, indicating more stereotyped inputs. We showed that reduced OFF excitation is due to the inhibition of OFF BC terminals, which is largely mediated by glycinergic inhibition. We also showed that the ON pathway significantly contributes to the inhibition of OFF RGCs, but not OFF BC terminals, and that gap junctions are not required to drive inhibitory outputs. ON/OFF cells displayed a proportional excitatory to inhibitory balance indicating that the ON and OFF layer inputs are activated equally by electrical stimulation in contrast to the asymmetry seen for ON and OFF RGCs separately. Finally, we showed that inhibition to OFF BCs may be abnormally strong in the *rd10* retina, causing a lower E/I balance in OFF cells in the *rd10* retina compared with those in the *wt* retina. Taken together, these results show that the consideration of how inhibitory circuits are activated by prosthetic electrical stimulation is necessary to guide rational stimulus design for vision restoration and that changes resulting from degeneration also need to be considered.

### Putative functional class grouping

Past work has shown that α ganglion cells show an asymmetry in E/I balance between some ON and OFF RGCs, similar to what we have shown for *rd10* RGCs here (Pang et al., 2003; Murphy and Rieke, 2006). Some evidence suggests this trend may extend to other RGC cell types (Roska et al., 2006), although E/I balance has yet to be systematically explored for all RGC subtypes. Our data for *wt* indicates that this general trend may hold true for a broad population of RGC subtypes. Our results also showed that ON/OFF RGCs have an equal E/I balance, consistent with other studies that have measured this, primarily in direction-selective ganglion cells (DSGCs; Poleg-Polsky and Diamond, 2016; Jain et al., 2020). In this work, instead of focusing on a subset of RGC types such as α ganglion cells, we attempted to sample across a large population of RGCs in an unbiased manner.

We performed whole-cell voltage–clamp recording and conductance analysis on 60 RGCs. As expected, this resulted in a population with heterogeneous response properties; however, the distribution of properties such as reversal potential was not unimodal. We identified two main modes of reversal potential consistent with conductance dominated by excitation and inhibition. In between these two modes was a weaker third mode. Although these three modes were not perfectly separable, we set boundary conditions at the troughs between modes (Fig. 3*A*) The individual groups resulting from this strategy were well separated, and the SDs of any one group did not overlap with the others (Figs. 3*C*, 10*C*). Although there were a small number of cells which lie close to the boundaries between modes and could potentially be misclassified, this only amounts to 3–6 cells out of 60 cells and excluding these cells does not change the conclusions of any subsequent analysis (data not shown).

Although we only filled a subset of cells (*n* = 12) to recover morphology, all the morphologies that we recovered matched the putative ON, OFF, and ON/OFF groups. We cannot be certain there are no deviations from this pattern in the entire sample; however, the majority of RGCs in each mode likely reflect the three distinct functional groups of RGCs. Importantly, the *wt* retina showed a similar multimodal distribution of reversal potential and E/I balance, albeit with a positive shifted E/I balance in the putative OFF group. Taken together, we believe that this work extends the basic finding of asymmetric E/I balance between ON and OFF RGCs to be a trend among many RGC subtypes rather than a subset. It also suggests that equal E/I ratio may be a general property of ON/OFF RGCs extending beyond DSGCs.

### The role of inhibition in prosthetic vision

Inhibition has many integral roles in vision formation (see above, Discussion) and has been shown to have a modulatory effect on degeneration-induced spontaneous activity (Margolis and Detwiler, 2011; Haq et al., 2014; Margolis et al., 2014; Carleton and Oesch, 2022), but the effect of electrical stimulation on inhibitory circuitry remains largely unexplored. Amacrine cells are the most diverse cell type of the retina with 60 identified cell types serving unique functions (Masland, 2012; Yan et al., 2020). These cells shape spatiotemporal responses to stimuli, create directional responses, and create center-surround responses, among a myriad of other integral mechanisms in creating vision; for reviews, see Diamond (2017) and Masland (2012). The field of retinal prosthetics has thus far focused on the generation of spikes with less attention on the underlying mechanisms that drive spiking during prosthetic stimulation (but see Walston et al., 2018). The inner nuclear layer is the main target of stimulation for subretinal prosthetics, and while BCs are depolarized in response to a stimulating current, it is also probable that amacrine cells are being stimulated as well (but see below). The interplay of simultaneous stimulation of amacrine and BCs is important in understanding how the degenerated circuit will respond to prosthetic stimulation. Prior work assessing the electrical stimulation of starburst amacrine cells identified that they were activated by inputs from presynaptic cells and not direct electrical stimulation; however, these studies were performed in the healthy rabbit retina, where photoreceptors and retinal thickness may impact the direct stimulation of the INL (Tsai et al., 2011; Cameron et al., 2013).

Previous studies have shown that electrical stimulation preferentially activates ON responses, although this has only been confirmed in studies on the healthy retina (Im and Fried, 2016; Lee and Im, 2019). Moreover, retinal prosthetic user reports are also consistent with preferential stimulation of the ON pathway (Humayun et al., 2003, 2012; Fujikado et al., 2007; Naycheva et al., 2012; Luo et al., 2016). How the true OFF pathway is recruited during retinal prosthetic stimulation is less certain, although OFF responses in some RGCs can be formed (Sekhar et al., 2017; Ho et al., 2018, 2020). Our finding that the OFF pathway receives significant inhibition during electrical stimulation may provide insight to past reports of poor OFF RGC responses to prosthetic stimulation; however, an explanation has not been previously put forth. The observation of altered inhibition in the *rd10* retina is consistent with general compensatory changes in inhibitory signaling shown in response to acute photoreceptor ablation (Care et al., 2020; Lee et al., 2022), although the specificity for the OFF versus ON pathway and presynaptic versus postsynaptic inhibition was not reflected in these studies. With a more complete understanding of the mechanisms driving electrical responses in ON and OFF RGCs, we may be able to better target stimulation that allows for pathway-specific activation.

## Synthesis

Reviewing Editor: Arvind Kumar, KTH Royal Institute of Technology Decisions are customarily a result of the Reviewing Editor and the peer reviewers coming together and discussing their recommendations until a consensus is reached. When revisions are invited, a fact-based synthesis statement explaining their decision and outlining what is needed to prepare a revision will be listed below. The following reviewer(s) agreed to reveal their identity: NONE. Note: If this manuscript was transferred from JNeurosci and a decision was made to accept the manuscript without peer review, a brief statement to this effect will instead be what is listed below.

Synthesis

In this manuscript the authors have characterized the effect of electrical stimulation of the retina. They show that electrical stimulation recruits the amount of excitation and inhibition received by On and Off cells is different. These results provide insights into the retinal circuitry and have relevance for retinal prosthetics. The manuscript was already reviewed (for J. Neurosci) and expectedly authors have already addressed much of the concerns raised by the reviewers. There are some issues that remain (see below) but that does not reduce the significance of results in the manuscript. The reviewers have identified a few point that either need a correction or clarification. Please see below for the reviewers' comments and submit a revised version of the manuscript along with the point-by-point reply.

Reviewer: 1

The paper by Carleton and Oesch studies the responses of retinal ganglion cells in the rd10 mouse to subretinal electrical stimulation, finding differences in the amounts of excitation and inhibition the cells receive. The results are largely consistent with previous work, extending the findings to the clinically relevant use of retinal stimulation. The paper has been previously reviewed and the authors have addressed these concerns, albeit in some cases more thorough responses would have led to an improved paper. For example, it is unfortunate that the morphological characterization was not done with field-standard methods, e.g., using confocal microscopy instead of epifluorescence. However, the results are worthy of publication and provide additional information to the field of retinal physiology that is relevant for vision prosthetics. My evaluation focuses on additional points of correction and clarification.

Points to address:

The statement in the abstract is slightly misleading because this is also the case in WT retina: "...in rd10 retina, we found electrically evoked synaptic inputs differed between ON and OFF RGC populations, with ON cells receiving mostly excitation and OFF cells receiving mostly inhibition and very little excitation". Do authors wish to revise?

The first sentence of the introduction is confusing. Is the point to undercut optogenetics or promote the Pixium Vision product? It does not serve to make the point the authors perhaps intend. Revision is needed.

Margolis et al. 2014 is very relevant to the interpretation of the work and should be cited either in the Introduction or Discussion. Given the amount of attention on the AII amacrine cell in the Results, this omission is surprising.

Margolis DJ, Gartland AJ, Singer JH, Detwiler PB. Network oscillations drive correlated spiking of ON and OFF ganglion cells in the rd1 mouse model of retinal degeneration. PLoS One. 2014 Jan 28;9(1):e86253. doi: 10.1371/journal.pone.0086253. PMID: 24489706; PMCID: PMC3904909.

Twyford et al. 2014 is extremely relevant and should be cited.

Twyford P, Cai C, Fried S. Differential responses to high-frequency electrical stimulation in ON and OFF retinal ganglion cells. J Neural Eng. 2014 Apr;11(2):025001. doi: 10.1088/1741-2560/11/2/025001. Epub 2014 Feb 21. PMID: 24556536; PMCID: PMC4465532.

In Methods, provide the age range of mice used. Great that age is plotted in Figure 9, but it would be useful to state age range in Methods.

Line 164, indicate type of microscope used. If not brand and model, at least specify epifluorescence, confocal, etc.

Line 166, indicate numerical boundaries used for ON/OFF definitions.

In Figure 3A, The black fit line seems to miss data points around 0 mV. The line also dips below 0 on the y-axis just before 0 mV. This makes no sense because there cannot be less than 0 cells in the histogram. This needs to be corrected.

Same as the previous issue for Figure 10A.

Reviewer: 2

The manuscript investigates the impact of electrical stimulation on the ex vivo degenerating (and wild-type control) mouse retina. The authors find that electrical stimulation engaged excitation and inhibition in systematically different ways among ON-type and OFF-type RGCs. This is convincingly demonstrated. While there are several caveats, such as the extent these results will generalize to human retinas and understanding/identifying the cell-type specific exceptions to the trends identified by the authors, the study and manuscript nevertheless advance the field of prosthetic development for the retina.

In my view, the authors have adequately addressed the comments raised by reviewers during the previous round of review and the manuscript is suitable for publication.

## Author Response

The statement in the abstract is slightly misleading because this is also the case in WT retina: "...in rd10 retina, we found electrically evoked synaptic inputs differed between ON and OFF RGC populations, with ON cells receiving mostly excitation and OFF cells receiving mostly inhibition and very little excitation". Do authors wish to revise? Author Response: We have now revised this sentence to include wt, while addressing the difference between the rd10 and wt later in the abstract (lines 50-51).

The first sentence of the introduction is confusing. Is the point to undercut optogenetics or promote the Pixium Vision product? It does not serve to make the point the authors perhaps intend. Revision is needed.

Author Response: We certainly did not intend to undercut the important work that is being done to assess optogenetics as a vision restoration technique or to specifically promote the Pixium prosthetic. Our intent was to point out that a retinal prosthetic device is the only vision restoration therapy currently in clinical trials for AMD. This device happens to be the Pixium device and therefore we included the citations for the Pixium Prima. We included the statement about optogenetics because it is an exciting new vision restoration technology that is often discussed along with retinal prosthetics; however, none- to our knowledge- are at the clinical trial stage for AMD. Thus, our goal was to underscore the importance of our work in that current clinical landscape. We appreciate the reviewer highlighting the fact that this could be perceived as a bias towards a particular technology on our part and have revised this introduction sentence to make these points more clearly.

Margolis et al. 2014 is very relevant to the interpretation of the work and should be cited either in the Introduction or Discussion. Given the amount of attention on the AII amacrine cell in the Results, this omission is surprising.

Margolis DJ, Gartland AJ, Singer JH, Detwiler PB. Network oscillations drive correlated spiking of ON and OFF ganglion cells in the rd1 mouse model of retinal degeneration. PLoS One. 2014 Jan 28;9(1):e86253. doi: 10.1371/journal.pone.0086253. PMID: 24489706; PMCID: PMC3904909.

Twyford et al. 2014 is extremely relevant and should be cited.

Twyford P, Cai C, Fried S. Differential responses to high-frequency electrical stimulation in ON and OFF retinal ganglion cells. J Neural Eng. 2014 Apr;11(2):025001. doi: 10.1088/1741-2560/11/2/025001. Epub 2014 Feb 21. PMID: 24556536; PMCID: PMC4465532.

Author Response: We are remiss that we did not include these references as they are indeed relevant to our discussion. We have now cited them where appropriate.

In Methods, provide the age range of mice used. Great that age is plotted in Figure 9, but it would be useful to state age range in Methods.

Author Response: While our methods initially stated our rd10 mice were older than p60 we have now corrected it to show the full age range, which was p62-p296.

Line 164, indicate type of microscope used. If not brand and model, at least specify epifluorescence, confocal, etc.

Author Response: Thank you we have now corrected this line to say we used an Olympus BX43 epifluorescence microscope.

Line 166, indicate numerical boundaries used for ON/OFF definitions.

Author Response: Our method of identifying the boundaries of the inner plexiform layer is similar to that of Margolis and Detwiler 2007, where the boundaries corresponded to 0 and 100%, and 50% was used as the delineation between the ON and OFF sublamina. We have now described these details in the text.

In Figure 3A, The black fit line seems to miss data points around 0 mV. The line also dips below 0 on the y-axis just before 0 mV. This makes no sense because there cannot be less than 0 cells in the histogram. This needs to be corrected.

Author Response: We thank the reviewer for allowing us to clarify this and correct the fit. We used the best fit of a multipeak gaussian to estimate the modes of the histogram, which is not constrained to pass through the data points. We have now held the baseline to 0 cells so there are no negative value approximations. This has been corrected in Figure 3A and 10A.

Same as the previous issue for Figure 10A.

Author Response: Please see above.

## References

[B1] Ames A, Nesbett FB (1981) In vitro retina as an experimental model of the central nervous system. J Neurochem 37:867–877. 10.1111/j.1471-4159.1981.tb04473.x 7320727

[B2] Anderson JR, et al. (2011) Exploring the retinal connectome. Mol Vis 17:355–379. PMCID: 21311605 PMC3036568

[B3] Borg-Graham LJ (2001) The computation of directional selectivity in the retina occurs presynaptic to the ganglion cell. Nat Neurosci 4:176–183. 10.1038/84007 11175879

[B4] Bourne RRA, et al. (2020) Global prevalence of blindness and distance and near vision impairment in 2020: progress towards the vision 2020 targets and what the future holds. Invest Ophthalmol Vis Sci 61:2317. https://iovs.arvojournals.org/article.aspx?articleid=2767477

[B6] Buldyrev I, Taylor WR (2013) Inhibitory mechanisms that generate centre and surround properties in on and off brisk-sustained ganglion cells in the rabbit retina. J Physiol 591:303–325. 23045347 10.1113/jphysiol.2012.243113 PMC3630787

[B5] Buldyrev I, Puthussery T, Taylor WR (2012) Synaptic pathways that shape the excitatory drive in an OFF retinal ganglion cell. J Neurophysiol 107:1795–1807. 10.1152/jn.00924.2011 22205648 PMC3331668

[B7] Cameron MA, Suaning GJ, Lovell NH, Morley JW (2013) Electrical stimulation of inner retinal neurons in wild-type and retinally degenerate (rd/rd) mice. PLoS ONE 8:e68882. 10.1371/journal.pone.0068882 23874798 PMC3708954

[B8] Care RA, Anastassov IA, Kastner DB, Kuo Y-M, Della Santina L, Dunn FA (2020) Mature retina compensates functionally for partial loss of rod photoreceptors. Cell Rep 31:107730. 10.1016/j.celrep.2020.107730 32521255 PMC8049532

[B9] Carleton M, Oesch NW (2022) Differences in the spatial fidelity of evoked and spontaneous signals in the degenerating retina. Front Cell Neurosci 16:1. 10.3389/fncel.2022.1040090 36419935 PMC9676928

[B10] Cook PB, McReynolds JS (1998) Lateral inhibition in the inner retina is important for spatial tuning of ganglion cells. Nat Neurosci 1:714–719. 10.1038/3714 10196588

[B12] Damle S, Lo Y-H, Freeman WR (2017) High visual acuity retinal prosthesis: understanding limitations and advancements toward functional prosthetic vision. Retina 37:1423–1427. 10.1097/IAE.0000000000001660 28426627

[B11] Damle S, Carleton M, Kapogianis T, Arya S, Cavichini-Corderio M, Freeman WR, Lo Y-H, Oesch NW (2021) Minimizing iridium oxide electrodes for high visual acuity subretinal stimulation. Eneuro 8:ENEURO.0506-20.2021. 10.1523/ENEURO.0506-20.2021 34799411 PMC8704424

[B13] Demb JB, Singer JH (2012) Intrinsic properties and functional circuitry of the AII amacrine cell. Vis Neurosci 29:51–60. 10.1017/S0952523811000368 22310372 PMC3561778

[B14] Diamond JS (2017) Inhibitory interneurons in the retina: types, circuitry, and function. Annu Rev Vis Sci 3:1–24. 10.1146/annurev-vision-102016-061345 28617659

[B15] Diamond JS, Lukasiewicz PD (2012) Amacrine cells: seeing the forest and the trees. Vis Neurosci 29:1–2. 10.1017/S0952523812000016 22416288

[B16] Drag S, Dotiwala F, Upadhyay AK (2023) Gene therapy for retinal degenerative diseases: progress, challenges, and future directions. Invest Ophthalmol Vis Sci 64:39. 10.1167/iovs.64.7.39 37389545 PMC10318594

[B17] Eggers ED, McCall MA, Lukasiewicz PD (2007) Presynaptic inhibition differentially shapes transmission in distinct circuits in the mouse retina. J Physiol 582:569–582. 10.1113/jphysiol.2007.131763 17463042 PMC2075342

[B18] Ellis EM, Paniagua AE, Scalabrino ML, Thapa M, Rathinavelu J, Jiao Y, Williams DS, Field GD, Fain GL, Sampath AP (2023) Cones and cone pathways remain functional in advanced retinal degeneration. Curr Biol 33:1513–1522.e4. 10.1016/j.cub.2023.03.007 36977418 PMC10133175

[B19] Farzad S, et al. (2023) Impact of retinal degeneration on response of ON and OFF cone bipolar cells to electrical stimulation. IEEE Trans Neural Syst Rehabil Eng 31:2424–2437. 10.1109/TNSRE.2023.3276431 37186528 PMC10324560

[B20] Flores-Herr N, Protti DA, Wässle H (2001) Synaptic currents generating the inhibitory surround of ganglion cells in the mammalian retina. J Neurosci 21:4852–4863. 10.1523/JNEUROSCI.21-13-04852.2001 11425912 PMC6762364

[B21] Franke K, Berens P, Schubert T, Bethge M, Nature TE (2017) Inhibition decorrelates visual feature representations in the inner retina. Nature 542:439–444. 28178238 10.1038/nature21394 PMC5325673

[B22] Fujikado T, et al. (2007) Evaluation of phosphenes elicited by extraocular stimulation in normals and by suprachoroidal-transretinal stimulation in patients with retinitis pigmentosa. Graefes Arch Clin Exp Ophthalmol 245:1411–1419. 10.1007/s00417-007-0563-z 17342502

[B23] Haq W, Arango-Gonzalez B, Zrenner E, Euler T, Schubert T (2014) Synaptic remodeling generates synchronous oscillations in the degenerated outer mouse retina. Front Neural Circuits 8:108. 10.3389/fncir.2014.00108 25249942 PMC4155782

[B25] Ho E, Smith R, Goetz G, Lei X, Galambos L, Kamins TI, Harris J, Mathieson K, Palanker D, Sher A (2018) Spatiotemporal characteristics of retinal response to network-mediated photovoltaic stimulation. J Neurophysiol 119:389–400. 10.1152/jn.00872.2016 29046428 PMC5867391

[B24] Ho E, Shmakov A, Palanker D (2020) Decoding network-mediated retinal response to electrical stimulation: implications for fidelity of prosthetic vision. J Neural Eng 17:066018. 33108781 10.1088/1741-2552/abc535 PMC8284336

[B26] Humayun MS, et al. (2003) Visual perception in a blind subject with a chronic microelectronic retinal prosthesis. Vision Res 43:2573–2581. 10.1016/S0042-6989(03)00457-7 13129543

[B27] Humayun MS, et al. (2012) Interim results from the international trial of second sight’s visual prosthesis. Ophthalmology 119:779–788. 10.1016/j.ophtha.2011.09.028 22244176 PMC3319859

[B28] Im M, Fried SI (2016) Temporal properties of network-mediated responses to repetitive stimuli are dependent upon retinal ganglion cell type. J Neural Eng 13:025002. 10.1088/1741-2560/13/2/025002 26905231 PMC4931047

[B29] Jain V, Murphy-Baum BL, deRosenroll G, Sethuramanujam S, Delsey M, Delaney KR, Awatramani GB (2020) The functional organization of excitation and inhibition in the dendrites of mouse direction-selective ganglion cells. eLife 9:583. 10.7554/eLife.52949 32096758 PMC7069718

[B30] Lee JY, Care RA, Kastner DB, Della Santina L, Dunn FA (2022) Inhibition, but not excitation, recovers from partial cone loss with greater spatiotemporal integration, synapse density, and frequency. Cell Rep 38:110317. 10.1016/j.celrep.2022.110317 35108533 PMC8865908

[B31] Lee J-I, Im M (2019) Optimal electric stimulus amplitude improves the selectivity between responses of ON versus OFF types of retinal ganglion cells. IEEE Trans Neural Syst Rehabil Eng 27:2015–2024. 10.1109/TNSRE.2019.2939012 31484127

[B32] Lee YT, Wang Q (1999) Inhibition of hKv2.1, a major human neuronal voltage-gated K+ channel, by meclofenamic acid. Eur J Pharmacol 378:349–356. 10.1016/S0014-2999(99)00485-9 10493112

[B33] Liang Z, Freed M (2010) The ON pathway rectifies the OFF pathway of the mammalian retina. J Neurosci 30:5533. 10.1523/JNEUROSCI.4733-09.2010 20410107 PMC3035477

[B34] Luo YH-L, Zhong JJ, Clemo M, da Cruz L (2016) Long-term repeatability and reproducibility of phosphene characteristics in chronically implanted argus II retinal prosthesis subjects. Am J Ophthalmol 170:100–109. 10.1016/j.ajo.2016.07.021 27491695

[B35] Manookin MB, Beaudoin DL, Ernst ZR, Flagel LJ, Demb JB (2008) Disinhibition combines with excitation to extend the operating range of the OFF visual pathway in daylight. J Neurosci 28:4136–4150. 18417693 10.1523/JNEUROSCI.4274-07.2008 PMC2557439

[B36] Marc RE, Jones BW, Watt CB, Strettoi E (2003) Neural remodeling in retinal degeneration. Prog Retin Eye Res 22:607–655. 10.1016/S1350-9462(03)00039-9 12892644

[B37] Margalit E, Thoreson WB (2006) Inner retinal mechanisms engaged by retinal electrical stimulation. Invest Ophthalmol Vis Sci 47:2606–2612. 10.1167/iovs.05-1093 16723477 PMC2474546

[B38] Margolis DJ, Detwiler PB (2007) Different mechanisms generate maintained activity in ON and OFF retinal ganglion cells. J Neurosci 27:5994–6005. 10.1523/JNEUROSCI.0130-07.2007 17537971 PMC3136104

[B39] Margolis DJ, Detwiler PB (2011) Cellular origin of spontaneous ganglion cell spike activity in animal models of retinitis pigmentosa. J Ophthalmol 2011:507037. 20936060 10.1155/2011/507037 PMC2948917

[B40] Margolis DJ, Gartland AJ, Singer JH, Detwiler PB (2014) Network oscillations drive correlated spiking of ON and OFF ganglion cells in the rd1 mouse model of retinal degeneration. PLoS ONE 9:e86253. 10.1371/journal.pone.0086253 24489706 PMC3904909

[B41] Margolis DJ, Newkirk G, Euler T, Detwiler PB (2008) Functional stability of retinal ganglion cells after degeneration-induced changes in synaptic input. J Neurosci 28:6526–6536. 10.1523/JNEUROSCI.1533-08.2008 18562624 PMC3050548

[B42] Masland RH (2001) The fundamental plan of the retina. Nat Neurosci 4:877–886. 10.1038/nn0901-877 11528418

[B43] Masland RH (2012) The tasks of amacrine cells. Vis Neurosci 29:3–9. 10.1017/S0952523811000344 22416289 PMC3652807

[B44] McLaughlin AJ, Percival KA, Gayet-Primo J, Puthussery T (2021) Glycinergic inhibition targets specific off cone bipolar cells in primate retina. eNeuro 8:1. 33188005 10.1523/ENEURO.0432-20.2020 PMC7920536

[B45] Molnar A, Hsueh H-A, Roska B, Werblin FS (2009) Crossover inhibition in the retina: circuitry that compensates for nonlinear rectifying synaptic transmission. J Comput Neurosci 27:569–590. 10.1007/s10827-009-0170-6 19636690 PMC2766457

[B46] Münch TA, da Silveira RA, Siegert S, Viney TJ, Awatramani GB, Roska B (2009) Approach sensitivity in the retina processed by a multifunctional neural circuit. Nat Neurosci 12:1308–1316. 10.1038/nn.2389 19734895

[B47] Murphy GJ, Rieke F (2006) Network variability limits stimulus-evoked spike timing precision in retinal ganglion cells. Neuron 52:511–524. 10.1016/j.neuron.2006.09.014 17088216 PMC2032021

[B48] Murphy GJ, Rieke F (2008) Signals and noise in an inhibitory interneuron diverge to control activity in nearby retinal ganglion cells. Nat Neurosci 11:318–326. 10.1038/nn2045 18223648 PMC2279192

[B49] Nagy J, Ebbinghaus B, Hoon M, Sinha R (2021) GABAA presynaptic inhibition regulates the gain and kinetics of retinal output neurons. 10.7554/eLife.60994 33904401 PMC8110304

[B50] Naycheva L, Schatz A, Röck T, Willmann G, Messias A, Bartz-Schmidt KU, Zrenner E, Gekeler F (2012) Phosphene thresholds elicited by transcorneal electrical stimulation in healthy subjects and patients with retinal diseases. Invest Ophthalmol Vis Sci 53:7440–7448. 10.1167/iovs.12-9612 23049087

[B51] Oesch NW, Diamond JS (2019) Synaptic inhibition tunes contrast computation in the retina. Vis Neurosci 36:35. 10.1017/S095252381900004X 31199207 PMC6578594

[B52] Oesch NW, Taylor WR (2010) Tetrodotoxin-resistant sodium channels contribute to directional responses in starburst amacrine cells. PLoS ONE 5:e12447. 10.1371/journal.pone.0012447 20805982 PMC2929195

[B53] Palanker D, Le Mer Y, Mohand-Said S, Muqit M, Sahel JA (2020) Photovoltaic restoration of central vision in atrophic age-related macular degeneration. Ophthalmology 127:1097–1104. 32249038 10.1016/j.ophtha.2020.02.024 PMC7384969

[B54] Pang J-J, Gao F, Wu SM (2003) Light-evoked excitatory and inhibitory synaptic inputs to ON and OFF alpha ganglion cells in the mouse retina. J Neurosci 23:6063–6073. 10.1523/JNEUROSCI.23-14-06063.2003 12853425 PMC6740343

[B55] Peretz A, Degani N, Nachman R, Uziyel Y, Gibor G, Shabat D, Attali B (2005) Meclofenamic acid and diclofenac, novel templates of KCNQ2/Q3 potassium channel openers, depress cortical neuron activity and exhibit anticonvulsant properties. Mol Pharmacol 67:1053–1066. 10.1124/mol.104.007112 15598972

[B56] Poleg-Polsky A, Diamond JS (2016) Retinal circuitry balances contrast tuning of excitation and inhibition to enable reliable computation of direction selectivity. J Neurosci 36:5861–5876. 10.1523/JNEUROSCI.4013-15.2016 27225774 PMC4879202

[B57] Pixium Vision SA (n.d.) *PRIMA US-Feasibility Study in Atrophic Dry AMD (PRIMA-FS-US)* (NCT03392324). Retrieved July 3, 2023, from https://www.clinicaltrials.gov/study/NCT03392324?intr=retinal%20prosthesis&aggFilters=status:rec%20act&rank=5

[B58] Puthussery T, Taylor WR (2010) Functional changes in inner retinal neurons in animal models of photoreceptor degeneration. Adv Exp Med Biol 664:525–532. 10.1007/978-1-4419-1399-9_60 20238055

[B59] Roska B, Molnar A, Werblin FS (2006) Parallel processing in retinal ganglion cells: how integration of space-time patterns of excitation and inhibition form the spiking output. J Neurophysiol 95:3810–3822. 10.1152/jn.00113.2006 16510780

[B60] Rubner R, Li KV, Canto-Soler MV (2022) Progress of clinical therapies for dry age-related macular degeneration. Int J Ophthalmol 15:157–166. 10.18240/ijo.2022.01.23 35047371 PMC8720345

[B61] Sagdullaev BT, McCall MA, Lukasiewicz PD (2006) Presynaptic inhibition modulates spillover, creating distinct dynamic response ranges of sensory output. Neuron 50:923–935. 10.1016/j.neuron.2006.05.015 16772173

[B62] Schiller PH (1992) The ON and OFF channels of the visual system. Trends Neurosci 15:86–92. 10.1016/0166-2236(92)90017-3 1373923

[B63] Sekhar S, Jalligampala A, Zrenner E, Rathbun DL (2017) Correspondence between visual and electrical input filters of ON and OFF mouse retinal ganglion cells. J Neural Eng 14:046017. 10.1088/1741-2552/aa722c 28489020

[B64] Slaughter MM, Miller RF (1983) An excitatory amino acid antagonist blocks cone input to sign-conserving second-order retinal neurons. Science 219:1230–1232. 10.1126/science.6131536 6131536

[B65] Stingl K, et al. (2015) Subretinal visual implant alpha IMS–clinical trial interim report. Vision Res 111:149–160. 10.1016/j.visres.2015.03.001 25812924

[B66] Stingl K, et al. (2017) Interim results of a multicenter trial with the new electronic subretinal implant alpha AMS in 15 patients blind from inherited retinal degenerations. Front Neurosci 11:e115239. 10.3389/fnins.2017.00445 28878616 PMC5572402

[B67] Taylor WR (1999) TTX attenuates surround inhibition in rabbit retinal ganglion cells. Vis Neurosci 16:285–290. 10.1017/S0952523899162096 10367963

[B68] Tian M, Jarsky T, Murphy GJ, Rieke F, Singer JH (2010) Voltage-gated Na channels in AII amacrine cells accelerate scotopic light responses mediated by the rod bipolar cell pathway. J Neurosci 30:4650–4659. 10.1523/JNEUROSCI.4212-09.2010 20357115 PMC3307391

[B69] Tsai D, Morley JW, Suaning GJ, Lovell NH (2011) Responses of starburst amacrine cells to prosthetic stimulation of the retina. *Conference Proceedings :… Annual International Conference of the IEEE Engineering in Medicine and Biology Society. IEEE Engineering in Medicine and Biology Society. Annual Conference*, 2011, 1053–1056. 10.1109/IEMBS.2011.6090245

[B70] Twyford P, Cai C, Fried S (2014) Differential responses to high-frequency electrical stimulation in ON and OFF retinal ganglion cells. J Neural Eng 11:025001. 10.1088/1741-2560/11/2/025001 24556536 PMC4465532

[B71] van Wyk M, Wässle H, Taylor WR (2009) Receptive field properties of ON- and OFF-ganglion cells in the mouse retina. Vis Neurosci 26:297–308. 10.1017/S0952523809990137 19602302 PMC2874828

[B72] Walston ST, Chow RH, Weiland JD (2018) Direct measurement of bipolar cell responses to electrical stimulation in whole-mount mouse retina. J Neural Eng 15:046003. 10.1088/1741-2552/aab4ed 29513646 PMC6657336

[B73] Wässle H (2004) Parallel processing in the mammalian retina. Nat Rev Neurosci 5:747–757. 10.1038/nrn1497 15378035

[B74] Weiland JD, Walston ST, Humayun MS (2016) Electrical stimulation of the retina to produce artificial vision. Ann Rev Vis Sci 2:273–294. 10.1146/annurev-vision-111815-114425 28532361

[B75] Yan W, Laboulaye MA, Tran NM, Whitney IE, Benhar I, Sanes JR (2020) Mouse retinal cell atlas: molecular identification of over sixty amacrine cell types. J Neurosci 40:5177–5195. 10.1523/JNEUROSCI.0471-20.2020 32457074 PMC7329304

